# Anti-Neuroinflammatory Effects of Prenylated Indole Alkaloids from the Antarctic Fungus *Aspergillus* sp. Strain SF-7367

**DOI:** 10.3390/molecules30020294

**Published:** 2025-01-13

**Authors:** Zhiming Liu, Chi-Su Yoon, Thao Quyen Cao, Hwan Lee, Il-Chan Kim, Joung Han Yim, Jae Hak Sohn, Dong-Sung Lee, Hyuncheol Oh

**Affiliations:** 1 Key Laboratory of Molecular Pharmacology and Drug Evaluation, Ministry of Education, School of Pharmacy, Yantai University, Yantai 264005, China; lzmqust@126.com; 2 Institute of Pharmaceutical Research and Development, College of Pharmacy, Wonkwang University, Iksan 54538, Republic of Korea; ycs1991@naver.com (C.-S.Y.); quyen.cao.thao@gmail.com (T.Q.C.); 3 Research Institute of Pharmaceutical Sciences (RIPS), College of Pharmacy, Chosun University, Dong-gu, Gwangju 61452, Republic of Korea; ghksdldi123@hanmail.net; 4 Division of Life Sciences, Korea Polar Research Institute, Incheon 21990, Republic of Korea; ickim@kopri.re.kr (I.-C.K.); jhyim@kopri.re.kr (J.H.Y.); 5 College of Medical and Life Sciences, Silla University, Busan 46958, Republic of Korea; jhsohn@silla.ac.kr

**Keywords:** *Antarctic fungi*, prenylated indole alkaloid, NF-κB, anti-inflammation, molecular docking

## Abstract

Inflammation has always been considered a trigger or consequence of neurodegenerative diseases, and the inhibition of inflammation in the central nervous system can effectively protect nerve cells. Several studies have indicated that various natural products inhibit neuroinflammation. Among these, Antarctic fungal metabolites have pharmacological activities and a developmental value. Therefore, this study aimed to evaluate the anti-neuroinflammatory activity of an Antarctic fungus belonging to *Aspergillus* (strain SF-7367). Secondary metabolites of SF-7367 were isolated using high-performance liquid chromatography followed by validation of their anti-inflammatory effects in lipopolysaccharide-stimulated BV2 microglia and RAW264.7 macrophages. Chemical analysis of metabolites from the fungal strain revealed five known compounds: epideoxybrevianamide E (**1**), brevianamide V/W (**2**), brevianamide K (**3**), brevianamide Q (**4**), and brevianamide R (**5**). Among these compounds, brevianamide K showed significant anti-inflammatory activity against both cell types. Results of Western blotting and molecular docking showed that brevianamide K could regulate the activation of nuclear factor kappa-light-chain-enhancer of activated B cell (NF-κB) signaling. This indicates that brevianamide K present in *Aspergillus* sp. (strain SF-7367) can inhibit inflammatory responses by reducing lipopolysaccharide-induced nuclear translocation of NF-κB (p65). These findings suggest that *Aspergillus* sp. (strain SF-7367) and brevianamide K are candidate agents for treating neurodegenerative diseases.

## 1. Introduction

Neuroinflammation is considered a cause or consequence of neurodegenerative diseases [1]. In an inflammatory environment, nerve cells undergo apoptosis because of the influence of inflammatory factors, leading to neurodegenerative diseases [2]. Therefore, researchers believe that controlling neuroinflammation is a strategy to delay the development and progression of neurodegenerative diseases [3]. Microglia are primary immune cells of the central nervous system. Similar to macrophages, microglia can transform into different phenotypes and balance the inflammatory response in the brain via pro- or anti-inflammatory mechanisms [4]. Thus, regulating microglial activation is key to regulating neuroinflammation.

There are many causes of neuroinflammation including viruses, bacteria, fungi, oxidative stress, and parasites [5]. Bacterial endotoxins are commonly used to induce neuroinflammation [6]. Lipopolysaccharide (LPS) is a common endotoxin that enters the nervous system and induces microglial activation [7]. Activated M1 microglia produce a large amount of pro-inflammatory factors, which trigger the promotion of inflammatory processes; inhibiting the activation of microglia is also considered to effectively inhibit neuroinflammation [8]. Nuclear factor kappa-light-chain-enhancer of activated B cells (NF-κB) is an important signaling pathway in neuroinflammation, and LPS can induce activation of the NF-κB pathway in microglia, leading to inflammation [9]. Within the NF-κB family, NF-κB p65 is an important subtype. Usually, p65 exists as a complex structure of p50 and NF-κB inhibitor alpha (IκB-α) in the cytoplasm, and LPS can cause phosphorylation of IκB-α, leading to the dissociation of p65 from the complex, and entry into the nucleus, binding to specific targets in the nucleus to promote transcription and production of pro-inflammatory factors [10]. Studies have also shown that inhibiting the nuclear transfer of p65 can suppress neuroinflammation [11,12,13].

*Antarctic fungi* are known for producing novel active components, and many studies have reported the presence of various compounds, such as polysaccharides, amino acids, flavonoids, and triterpenes as secondary metabolites [14,15,16]. Many of these compounds have pharmacological activities, and whether they can be used to treat neurodegenerative diseases is worth exploring. In the present study, we isolated five prenylated indole alkaloids from the Antarctic fungus SF-7367 and validated their anti-inflammatory activities in LPS-stimulated BV2 microglia and RAW264.7 macrophages. We also validated the regulatory ability of the active compound on NF-κB activation, thus preliminarily exploring the anti-neuroinflammatory effects of the secondary metabolites of the Antarctic fungus SF-7367.

## 2. Results

### 2.1. Isolation and Structure Determination of Five Metabolites

In our continuing study on the chemical components of *Antarctic fungi*, five brevianamide metabolites were isolated from ethyl acetate extracts of SF7367 using various chromatographic methods, including column chromatography and HPLC.

**Figure 1:**
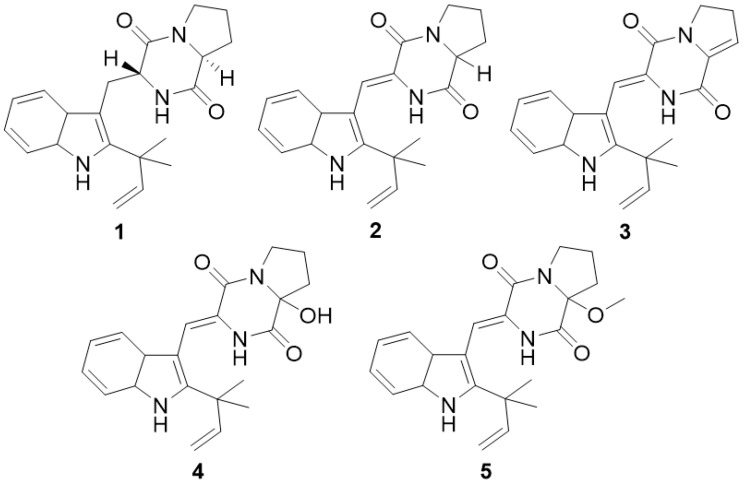
Chemical structures of the five compounds (**1**–**5**) isolated from the Antarctic fungus *Aspergillus* sp. strain SF-7367.

The ^1^H NMR data (Supplementary Figures S1–S15) showed all five compounds having similar chemical shift patterns. The chemical shift value presented around 7.0 to 7.5 ppm suggested the existence of the benzene moiety in the indole ring; in addition, the presence of an isoprenylated group was observed near 5.1 and 6.2 ppm. The greatest difference in the chemical shift value for all compounds was observed at the diketopiperazine ring moiety, such as the presence of hydroxyl- and methoxyl-groups and the presence or absence of double bond. However, the spectroscopic data for compounds **2**, **4**, and **5** were not sufficient to figure out the absolute configuration of the proline ring; therefore, their chemical structures remained as a racemic mixture. Based on a comparison of their MS and NMR data with those previously reported in the literature, they were identified as epi-deoxybrevianamide E (**1**), brevianamide K (**3**), and three racemic mixture components: brevianamide V/W (**2**), brevianamide Q (**4**), and brevianamide R (**5**) (Figure 1).

### 2.2. Anti-Inflammatory Effects of Five Metabolites in LPS-Stimulated BV2 and RAW264.7 Cells

**Figure 2:**
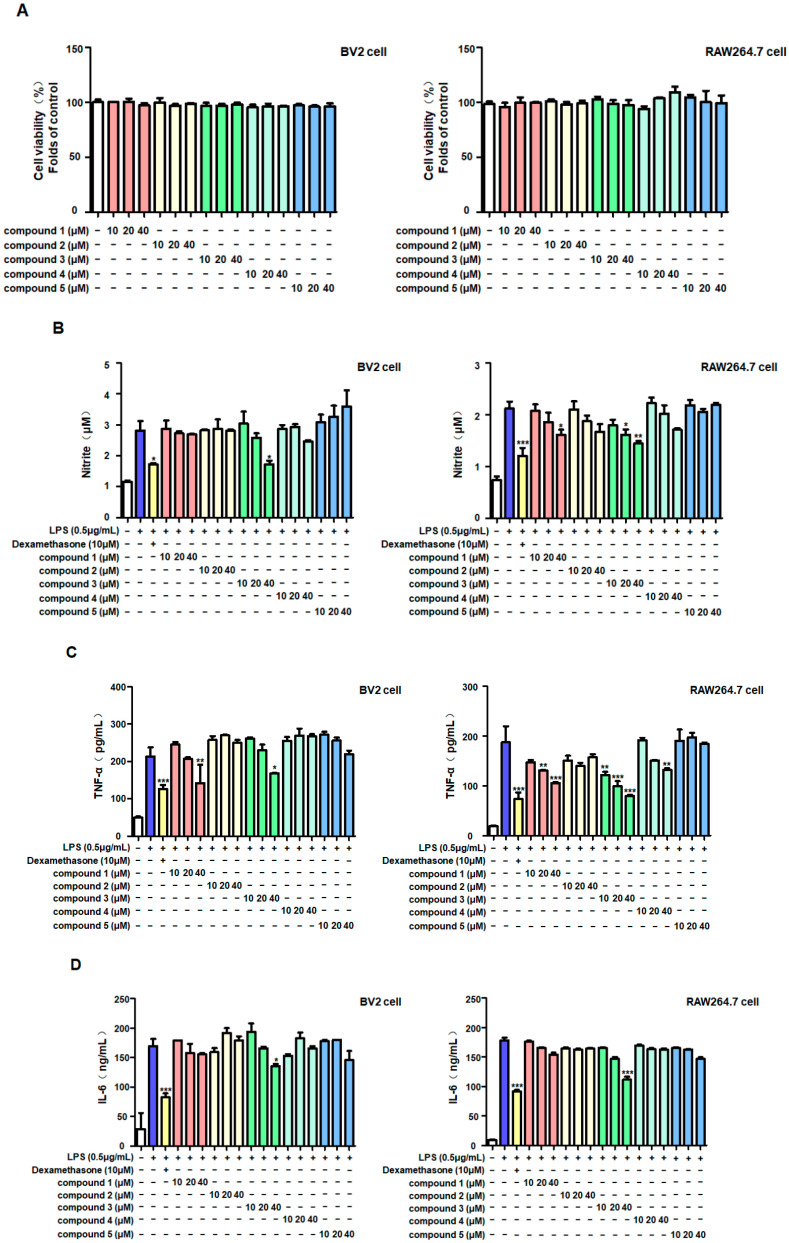
The inhibitory effect of the five compounds on inflammatory factors. After incubating BV2 cells and RAW264.7 cells with different concentrations of compounds for 24 h, the cytotoxicity of the five compounds was determined using the MTT assay (**A**). Then, cells were pretreated with compounds within a safe concentration range for 2 h, followed by induction of cell inflammation with LPS (0.5 μg/mL). After 24 h, the concentrations of nitrite (**B**), tumor necrosis factor (TNF)—α (**C**), and interleukin-6 (**D**) in the supernatant of both types of cells were measured. Bars represent means ± standard deviation of three independent experiments. * p < 0.05, ** p < 0.01, *** p < 0.001 compared with the LPS group.

In this study, we evaluated the potential anti-neuroinflammatory activities of these five compounds. The MTT method was used to evaluate the cytotoxicity of the compounds. The results showed that the five compounds did not exhibit significant cytotoxicity on BV2 and RAW264.7 cells within the pre-set concentration range (10–40 μM) (Figure 2A). Therefore, this concentration range was used in the subsequent experiments. The inhibitory effects of the five compounds on inflammatory factors were tested using LPS to induce inflammation in the two cell types, and dexamethasone was used as a positive control. The results showed that only brevianamide K (**3**) significantly inhibited nitrite production in both cell types simultaneously (Figure 2B). In addition, brevianamide K (**3**) could also reduce the excessive production of TNF-α and IL-6 induced by LPS (Figure 2C,D).

**Figure 3:**
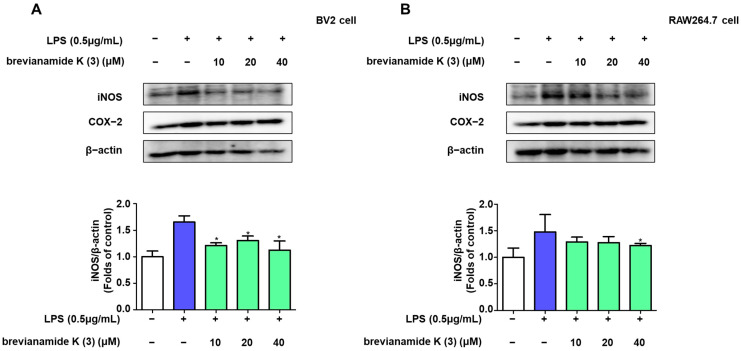
Suppressing effects of brevianamide K (**3**) on iNOS expression. BV2 cells and RAW264.7 cells were pretreated with different concentrations of brevianamide K (**3**) for 2 h, followed by induction of cell inflammation with LPS (0.5 μg/mL). After 24 h, the expression of iNOS in BV2 (**A**) or RAW264.7 cell (**B**) was determined using Western blotting. Representative blots from three independent experiments are shown. Band intensities were analyzed using ImageJ software_v1.8.0. Bars represent means ± standard deviation of three independent experiments. * p < 0.05 compared with the LPS group.

Western blotting was performed to determine the anti-inflammatory mechanism of brevianamide K (**3**). Liposaccharide upregulated the expression of the inflammatory proteins inducible NOS (iNOS) in both cell types (Figure 3). Brevianamide K (**3**) selectively reduced iNOS expression.

### 2.3. Inhibitory Effect of Brevianamide K (***3***) on NF-κB Activation

**Figure 4:**
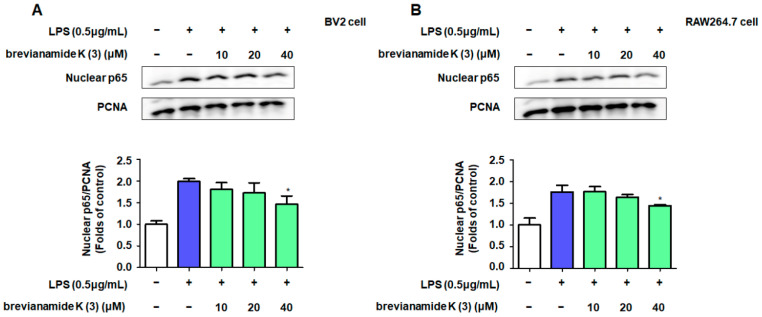

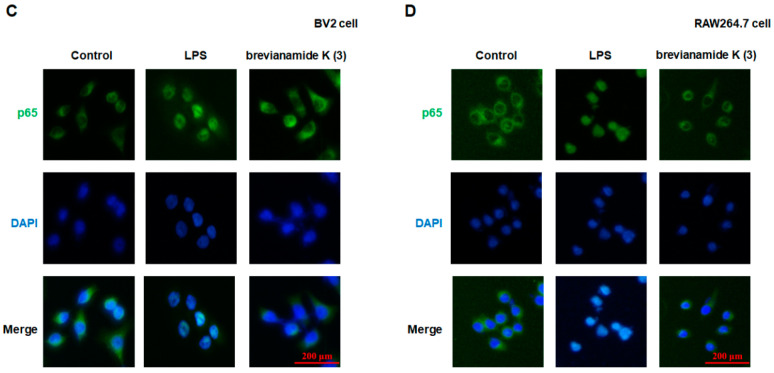
Suppressing effects of brevianamide K (**3**) on NF-κB activation. BV2 cells and RAW264.7 cells were pretreated with different concentrations of brevianamide K (**3**) for 2 h, followed by the induction of cell inflammation with LPS (0.5 μg/mL). After 0.5 h, the nuclear fraction was separated according to the instructions of the kit, the expression of nuclear p65 was determined using Western blotting. Representative blots from three independent experiments are shown (**A**). Band intensities were analyzed using ImageJ software (**B**). Localization of p65 in BV2 (**C**) and RAW264.7 cells (**D**) detected by the immunofluorescence assay. Bars represent means ± standard deviation of three independent experiments. * p < 0.05 compared with the LPS group.

To further confirm the anti-inflammatory mechanism of brevianamide K (**3**), we tested the effect of brevianamide K (**3**) on nuclear translocation of NF-κB (p65). Western blotting results showed that p65 expression increased in the nucleus of LPS-treated cells compared to that in untreated cells, whereas brevianamide K (**3**) inhibited this increase (Figure 4A,B). The immunofluorescence results showed a similar trend. In normal cells, p65 was more concentrated in the cytoplasm, whereas in cells co-cultured with LPS, p65 fluorescence was more concentrated in the nucleus. Treatment with brevianamide K (**3**) prevented nuclear translocation of p65 (Figure 4C,D).

### 2.4. Role of NF-κB Activation in the Inflammation Inhibitory Effect of Brevianamide K (***3***)

**Figure 5:**
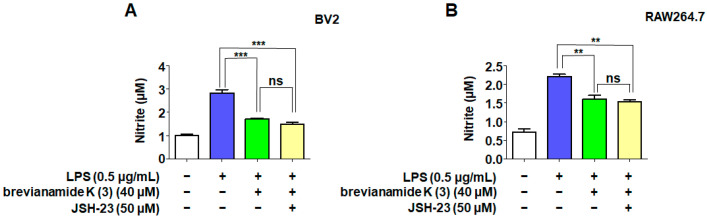

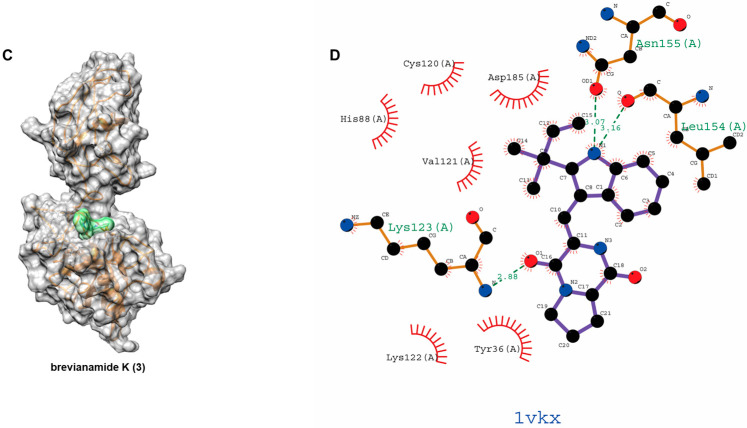
Anti-inflammatory effects of brevianamide K (**3**) related to the p65 in NF-κB signaling pathway. BV2 cells and RAW264.7 cells were pretreated with JSH-23 or different concentrations of brevianamide K (**3**) for 2 h, followed by induction of cell inflammation with LPS (0.5 μ g/mL). After 24 h, the concentrations of nitrite in the supernatant of BV2 (**A**) and RAW264.7 cell (**B**) were measured. Bars represent means ± standard deviation of three independent experiments. ** p < 0.01, *** *p* < 0.001 compared to the LPS group. ns > 0.05 compared with the brevianamide K (**3**)-treated group. Molecular docking of SF7367-3-10 in the NF-κB (p65) protein (PDB ID: 1VKX) was determined using Chimera 1.15 (**C**), and the covalent binding site of brevianamide K (**3**) to residues of p65 is displayed. The interaction between the two was analyzed using LIGPLOT software (**D**). The dotted line (green) indicates potential peptide bonding interactions between representative amino acid residues in the binding pocket of p65.

To verify whether brevianamide K (**3**) exerts anti-inflammatory effects by acting on NF-κB, we used JSH-23, an inhibitor of p65 nuclear transcription, to compare the anti-inflammatory effects of brevianamide K (**3**). The results showed that brevianamide K (**3**) significantly inhibited nitric oxide production in both cell types (Figure 5A,B). However, when the cells were co-treated with JSH-23 and brevianamide K (**3**), the production of nitrate was not significantly reduced compared to when using compound 3 alone, indicating that the two drugs did not have a synergistic inhibitory effect on nitrite production. Molecular docking was performed to verify the possible binding mode between brevianamide K (**3**) and p65. Figure 5C shows the optimal binding conformation between brevianamide K (**3**) and p65. The interaction between the two was analyzed using LIGPLOT v.2.2.5 software (Figure 5D). The results showed that three peptide bonds (green dashed line) were present between brevianamide K (**3**) and the three amino acid residues Lys123 (A), Asn155 (A), and Leu154 (A).

**Figure 6:**
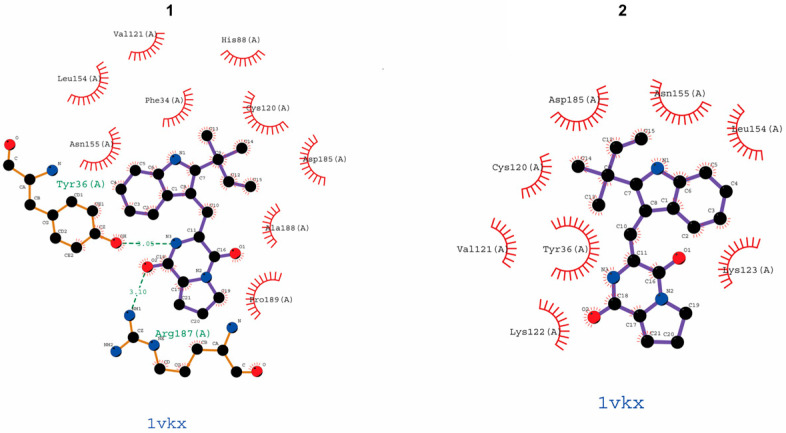

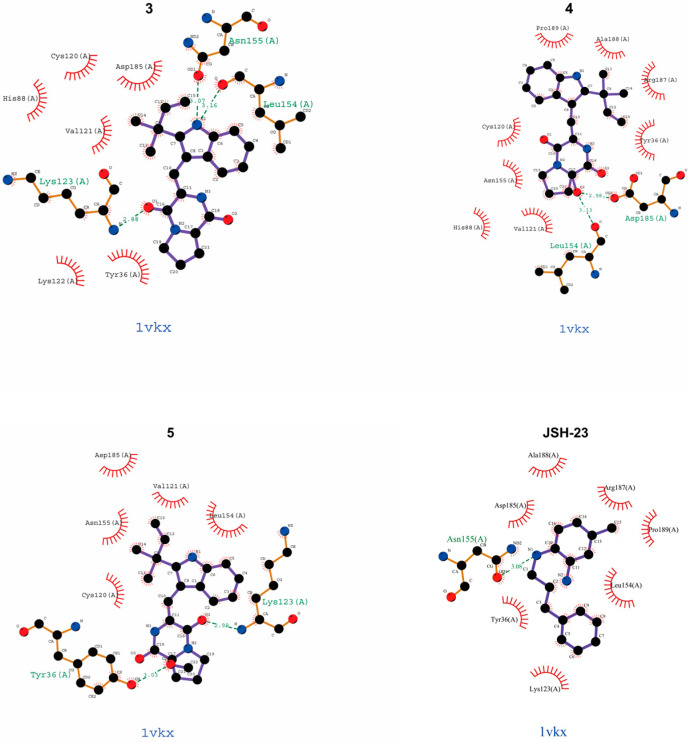
Molecular docking of five compounds and JSH-23 in the NF-κB (p65) protein. Docking was determined using Chimera 1.15, and the covalent binding site of the compounds to residues of p65 is displayed. The interaction between the two was analyzed using LIGPLOT software. The dotted line (green) indicates potential peptide bonding interactions between representative amino acid residues in the binding pocket of p65.

Due to the effect of brevianamide K (**3**) on p65 and its anti-inflammatory effect. Molecular docking was used to predict the interaction between the other four compounds and JSH-23 with p65 (Figure 6). After analysis, except for compound **2**, which did not have peptide bonding with the pocket of p65, all other compounds had at least one peptide bond with p65. Among them, JSH-23 formed a peptide bond with the amino acid residue Asn155 (A), and among the five SF7367 compounds, only brevianamide K (**3**) also acted on Asn155 (A) (Table 1).

## 3. Discussion

Prenylated indole alkaloids containing indole/indoline and isoprenoid moieties display broad structural diversity and are components of the genera *Penicillium* and *Aspergillus* of Ascomycota [17]. These diverse chemical structures have been reported as having a wide range of biological and pharmacological activities [18]. Tryptophan is considered a key precursor that acts as a biogenetic source of indole or indoline rings [19]. In addition, most of these structures are cyclic dipeptides with a diketopiperazine structure or its derivative [20]. In the present study, we investigated the bioactivity of five reverse-prenylated indole alkaloids with diketopiperazines. Although these alkaloids have exceedingly similar structures, the results showed different biological effects. Brevianamide K (**3**) showed the strongest anti-inflammatory effects, and its structural difference from other compounds was the existence of a double bond in the proline moiety of the diketopiperazine ring.

Neuroinflammation is widely believed to be the result or trigger of neurodegenerative diseases, and long-term inflammatory environments may lead to the permanent deterioration of neurodegenerative processes [21]. Inhibition of excessive inflammatory responses is considered an important treatment strategy for protecting the nervous system [22]. Thus, the role of nitrite in the nervous system cannot be ignored. As a common cellular mediator, nitrite plays different roles under different situations [23]. Upon endotoxin stimulation, nitrite is produced by microglia and macrophages [24,25]. Nitrite is a pro-inflammatory factor that can exacerbate inflammation, damage the neuronal cell membrane structure, and affect DNA transcription and synthesis [26]. Our results showed that under the stimulatory effect of the endotoxin LPS, both BV2 and RAW264.7 immune cells produced high concentrations of nitrite, whereas brevianamide K (**3**) exhibited significant anti-inflammatory effects. According to previous studies, LPS-stimulated BV2 and RAW264.7 cells also produce other important pro-inflammatory mediators, such as TNF-α and IL-6 [27,28]. The production of large amounts of TNF-α and IL-6 in immune cells is an important factor in causing acute brain inflammation [29]. Similarly, brevianamide K (**3**) suppressed excessive production of these inflammatory factors (Figure 2). This indicates that brevianamide K (**3**) effectively inhibits the production of inflammatory factors caused by endotoxins. The role of certain enzymes in the production of these inflammatory factors cannot be ignored. According to previous reports, iNOS is usually not expressed in cells, whereas LPS can enhance the expression of iNOS in immune cells, thereby accelerating the production and release of nitrites [30,31]. Western blotting showed that brevianamide K (**3**) inhibited iNOS expression in both cell types (Figure 3A,B). In our previous studies, the expression of iNOS showed consistent trends [32,33]; therefore, we speculate that this was because brevianamide K (**3**) selectively inhibited the activity of the iNOS promoter.

NF-κB is a promoter of iNOS and serves as a key inflammatory regulator [34]. Activation of NF-κB signaling is crucial for the progression of neuroinflammation. RelA (p65) is a key subtype of NF-κB and a nuclear localization signal. Owing to the presence of the IκB protein, p65 is usually stably present in the cytoplasm [35]. This is because IκB-α can interact with the p65 protein to induce a helix conformation. However, under LPS stimulation, IκB-α is phosphorylated, causing p65 to dissociate from the alpha helix conformation and transfer to the nucleus, binding to targets on DNA strands and promoting transcription of inflammatory factors [36]. Western blotting showed that brevianamide K (**3**) significantly reduced the protein levels of nuclear p65 in BV2 and RAW264.7 cells (Figure 4A,B). To observe the position of p65 more intuitively, we used immunofluorescence to locate p65 in these two cell types. The results confirmed that LPS promotes the nuclear translocation of p65, whereas brevianamide K (**3**) inhibits the nuclear translocation of p65 in both cell types. This indicates that brevianamide K (**3**) can effectively inhibit the activation of the NF-κB pathway. JSH-23 is an inhibitor of p65 nuclear translocation. After simultaneous treatment of the cells with JSH-23 and brevianamide K (**3**), the two drugs did not show synergistic effects on nitrite production (Figure 5A,B), indicating that both samples exerted anti-inflammatory effects by inhibiting p65 nuclear translocation. To investigate the structure–activity relationship of the compound, we used molecular docking to evaluate the ability of brevianamide K (**3**) to bind to the p65 receptor. Figure 5C shows the optimal interaction mode between brevianamide K (**3**) and the p65 receptor. Subsequently, we analyzed the binding sites under the optimal interaction mode using LIGPLOT software, as shown in Figure 5D. Three peptide bonds were present between brevianamide K (**3**) and the p65 receptor. This enabled brevianamide K (**3**) to bind tightly to the p65 receptor site, indirectly confirming the pharmacological activity of the compound by acting on p65, except for brevianamide K (**3**). Molecular docking was used to predict the interaction between the other four compounds and JSH-23 with p65 (Figure 6). After analysis, except for compound 2, which did not have peptide bonding with the pocket of p65, all other compounds had at least one peptide bond with p65. JSH-23 formed a peptide bond with the amino acid residue Asn155 (A). Among the five compounds, only brevianamide K (**3**) also acted on Asn155 (A), which to some extent confirms that brevianamide K (**3**) may compete with JSH-23 for binding to the pocket of the p65 protein. The reason brevianamide K (**3**) exhibits the best anti-inflammatory effect among evaluated *Antarctic fungi* may be owing to the unique spatial structure of brevianamide K (**3**), which allows the compound to inhibit the activation of the NF-κB pathway highly effectively.

## 4. Materials and Methods

### 4.1. General Experimental Procedures

HRESI-MS data were obtained using a Q-TOF micro-LC-MS/MS instrument (Waters, Manchester, UK). The optical rotations were recorded using a Jasco p-2000 digital polarimeter. NMR spectra (1D- and 2D-NMR) were recorded in methanol-*d*_4_ with a JEOL JNM ECP-400 spectrometer (JEOL Ltd., Akishima, Japan), and the chemical shifts were referenced relative to the residual solvent peaks (methanol-*d*_4_: *δ*_H_/*δ*_C_ = 3.31/49.00). HSQC and HMBC experiments were optimized for ^1^*J*_CH_ = 140 Hz and ^n^*J*_CH_ = 8 Hz, respectively. HPLC (YOUNGLIN-YL9100, Younglin, Anyang, Korea) separation was performed using a SHISEIDO CAPCELL PAK C18 column (20 mm × 150 mm, 5 μm particle size, SHISEIDO Co., Tokyo, Japan) at a flow rate of 5 mL/min, and the solvents used for HPLC were of analytical grade. Roswell Park Memorial Institute (RPMI) 1640 medium, Dulbecco’s modified Eagle’s medium, fetal bovine serum (FBS), and other tissue culture reagents were purchased from Gibco BRL Co. (Grand Island, NY, USA). All other chemicals were purchased from Sigma-Aldrich (St. Louis, MO, USA).

### 4.2. Fungal Material

*Aspergillus* sp. (strain SF7367) was isolated from calcareous algae that was collected at the Barton Peninsula, Antarctica in February 2017. The fungal strain SF-7367 was identified based on ITS gene sequence analysis. A GenBank search of the ITS gene of SF-7367 (GenBank accession number OP216728) indicated *Aspergillus jensenii* (NR_135444), *A. tennesseensis* (NR_135447), *A. versicolor* (NR_131277), *A. protuberus* (NR_135353), and *A. creber* (NR_135442) as the closest matches, with sequence identities of 100%, 100%, 100%, 100%, and 99.81%, respectively. Therefore, the fungal strain SF-7367 was identified as *Aspergillus* sp.

### 4.3. Fungal Culture, Extraction, and Isolation

The fungal strain SF7367 was cultured in 20 Fernbach flasks, each containing 150 mL of potato dextrose agar medium with 3% NaCl (*v*/*v*). The flasks were individually inoculated with 2 mL of seed culture of the fungal strain and incubated at 25 °C for 14 d. The fermented culture media were combined and extracted with EtOAc (20 L). The combined EtOAc extracts were filtered through filter paper and evaporated to dryness, resulting in a crude extract (2.0597 g). The crude extract of SF7367 was fractionated by reversed-phase C_18_ flash column chromatography (5.5 × 30 cm) and eluted with a stepwise gradient of 20%, 40%, 60%, 80%, and 100% (*v*/*v*) MeOH in H_2_O (500 mL each) to obtain five sub-fractions, SF7367-1 to SF7367-5. Fraction SF7367-3 (119.8 mg) was separated by C_18_ prep HPLC (eluted with a gradient solvent system of 25–60% CH_3_CN in H_2_O, over 55 min) to obtain compounds **1** (8.4 mg, t_R_ = 38 min), **2** (3.2 mg, t_R_ = 39 min), **3** (3.2 mg, t_R_ = 41 min), and sub-fractions SF7367-3-1–7 and SF7367-3-12. The subfraction SF7367-3-12 (5.9 mg) was purified using C_18_ prep HPLC (eluted with a gradient solvent system of 40–60% CH_3_CN in H_2_O, over 60 min) to obtain **4** (1.1 mg, t_R_ = 17 min) and **5** (69.6 mg, t_R_ = 31 min). The NMR and MS data for all compounds are in Supplementary Figures S1–S15.

### 4.4. Cell Culture and Viability

BV2 and RAW264.7 cells were cultured in an RPMI1640 medium (containing 10% FBS and 1% penicillin/streptomycin). To test the toxicity of the compounds, the two cell types were inoculated into 48 well plates at a cell density of 5 × 10^4^ cells/well. After overnight cell cultivation, cells were treated with different concentrations of the test compounds for 24 h and then treated with 3-(4,5)-dimethylthiahiazo (-z-y1)-3,5-diphenyltetrazolium bromide (MTT) solution (5 mg/mL) for 30 min. The supernatant was then removed, the purple methylzan crystals were dissolved in the culture plate well with dimethyl sulfoxide, and the absorbance was measured at 540 nm [32].

### 4.5. Determination of Nitrite Levels

The cell supernatant was mixed with Griess reagent at a 1:1 ratio, and the absorbance was measured at 570 nm. Sodium nitrite was used as a standard control solution to determine the concentration of nitrate in the culture medium [33].

### 4.6. Determination of Inflammatory Factor Levels

ELISA was used to detect concentrations of the inflammatory factors TNF-α, IL-6, and PGE_2_ in the BV2 and RAW264.7 cell supernatants according to the manufacturer’s instructions.

### 4.7. Western Blot Analysis

RIPA buffer containing a phosphatase inhibitor was used to lyse cells in a 6-well plate. Cytosolic and nuclear fractions were separated according to the manufacturer’s instructions (Cayman, Ann Arbor, MI, USA). After centrifugation, the supernatant was used for the subsequent experiments. SDS-PAGE was used for Western blotting, and a nitrocellulose membrane was used to transfer proteins onto the gel. The nitrocellulose membrane was sealed with 5% skim milk powder solution for 1 h, followed by incubation with primary antibodies overnight for 4 h. Next, secondary antibodies were added, and the cells were incubated for 1 h. The culture was washed three times with TBST, and the antibody solution was changed each time. An ECL solution was used to treat the nitrocellulose membrane, which was placed in an imaging device for development. ImageJ software was used to determine the optical densities of the obtained bands.

### 4.8. Synergistic Effect Detection of JSH-23 (p65 Nuclear Inhibitor)

BV2 cells and RAW264.7 cells were pretreated with JSH-23 (50 nM) [37] or different concentrations of brevianamide K (**3**) for 2 h, followed by the induction of cell inflammation with LPS (0.5 μg/mL). After 24 h, the concentrations of nitrite in the supernatant of BV2 (A) and RAW264.7 (B) cells were measured.

### 4.9. Molecular Docking

Molecular docking was performed to predict the possible binding site of SF7367 on the p65 protein using AutoDock Vina. The crystal structure of p65 (PDB ID:1VKX) was obtained from the RCSB database (https://www.rcsb.org/ (accessed on 20 September 2024)). The 3D structure of brevianamide K (**3**) obtained using MarvinSketch software (version 20.7.0, http://www.chemaxon.com (accessed on 20 September 2024)) was used for docking. The binding sites for p65 were Arg33, Arg35, Tyr36, Cys38, Glu39, Lys122, Lys123, Arg187, Lys218, Gln220, Lys221, Arg246, and Gln247 [38]. Modeling studies were performed using UCSF Chimera software (version 1.15, https://www.cgl.ucsf.edu/chimera/ (accessed on 20 September 2024)). Potential peptide bonds and van der Waals contacts in the lowest binding energy docking model were analyzed using LIGPLOT^+^ software (version v.2.2.5).

### 4.10. Statistical Analysis

Data were analyzed using GraphPad Prism 5.01 software, and significant differences were analyzed using Tukey’s one-way analysis of variance. Data are presented as the mean and variance of at least three independent experiments.

## 5. Conclusions

In this study, we isolated and identified five reverse-prenylated indole alkaloids from the Antarctic fungus *Aspergillus* sp. (strain SF-7367). Among these compounds, brevianamide K (**3**) was the only compound that significantly inhibited the inflammatory response caused by the endotoxin LPS, which was related to its inhibitory function on NF-κB signaling. This also demonstrated that the functional groups at the N-1 and O-16 positions of the compound may be the only key structural features with significant anti-inflammatory effects. This study aimed to provide the potential of brevianamide K (**3**) as a therapeutic agent for neurodegenerative disease. However, because of limitations in the sample size and experimental conditions, further research or animal experiments may be needed to reveal a clear structure–activity relationship in brevianamide K (**3**).

## Figures and Tables

**Table 1:** The binding sites of peptide bonds between compounds and the p65 protein.

Compound	Binding Site (Amino Acid Residues of p65)
epi-deoxybrevianamide E (**1**)	Try16 (A), Arg187 (A)
brevianamide V/W (**2**)	**−**
brevianamide K (**3**)	Asn155 (A), Leu154 (A), Lys123 (A)
brevianamide Q (**4**)	Leu154 (A), Asp185 (A)
brevianamide R (**5**)	Try36 (A), Lys123 (A)
JSH-23	Asn155 (A)

## Data Availability

The data presented in this study are available on request from the corresponding author.

## Supplementary Materials

The following supporting information can be downloaded at: https://www.mdpi.com/article/10.3390/molecules30020294/s1. The NMR and MS spectrum data for all compounds are in a Supplementary File. Figure S1. ^1^H NMR spectrum (methanol-*d_4_*) of epideoxybrevianamide E (**1**). Figure S2. ^13^C NMR spectrum (methanol-*d_4_*) of epideoxybrevianamide E (**1**). Figure S3. HRESI-MS spectrum of epideoxybrevianamide E (**1**). Figure S4. ^1^H NMR spectrum (methanol-*d_4_*) of brevianamide V/W (**2**). Figure S5. ^13^C NMR spectrum (methanol-*d_4_*) of brevianamide V/W (**2**). Figure S6. HRESI-MS spectrum of brevianamide V/W (**2**). Figure S7. ^1^H NMR spectrum (methanol-*d_4_*) of brevianamide K (**3**). Figure S8. ^13^C NMR spectrum (methanol-*d_4_*) of brevianamide K (**3**). Figure S9. HRESI-MS spectrum of brevianamide K (**3**). Figure S10. ^1^H NMR spectrum (methanol-*d_4_*) of brevianamide Q (**4**). Figure S11. ^13^C NMR spectrum (methanol-*d_4_*) of brevianamide Q (**4**). Figure S12. HRESI-MS spectrum of brevianamide Q (**4**). Figure S13. ^1^H NMR spectrum (methanol-*d_4_*) of brevianamide R (**5**). Figure S14. ^13^C NMR spectrum (methanol-*d_4_*) of brevianamide R (**5**). Figure S15. HRESI-MS spectrum of brevianamide R (**5**).
