# Supplementary material for: Anti-Neuroinflammatory Effects of Prenylated Indole Alkaloids from the Antarctic Fungus Aspergillus sp. Strain SF-7367
Source: Molecules. 2025 Jan 13;30(2):294. doi: 10.3390/molecules30020294 (PMC11767326; doi:10.3390/molecules30020294)
Supplement: Supplementary file 1 [file molecules-30-00294-s001.zip › molecules-3316657-supplementary.pdf]

Anti-Neuroinflammatory Effects of Prenylated Indole Alkaloids from the Antarctic Fungus *Aspergillus* sp. Strain SF-7367

Zhiming Liu [1,†], Chi-Su Yoon [2,†], Thao Quyen Cao [2], Hwan Lee [3], Il-Chan Kim [4], Joung Han Yim [4], Jae Hak Sohn [5], Dong-Sung Lee [3,*], and Hyuncheol Oh [2,*]

[1] Key Laboratory of Molecular Pharmacology and Drug Evaluation, Ministry of Education, School of Pharmacy, Yantai University, Yantai 264005, China; lzmqust@126.com
[2] Institute of Pharmaceutical Research and Development, College of Pharmacy, Wonkwang University,
Iksan 54538, Republic of Korea; ycs1991@naver.com (C.-S.Y.); quyen.cao.thao@gmail.com (T.Q.C.)
[3] Research Institute of Pharmaceutical Sciences (RIPS), College of Pharmacy, Chosun University,
Dong-gu, Gwangju 61452, Republic of Korea; ghksdldi123@hanmail.net
[4] Division of Life Sciences, Korea Polar Research Institute, Incheon 21990, Republic of Korea; ickim@kopri.re.kr (I.-C.K.); jhyim@kopri.re.kr (J.H.Y.)
[5] College of Medical and Life Sciences, Silla University, Busan 46958, Republic of Korea; jhsohn@silla.ac.kr
* Correspondence: dslee2771@chosun.ac.kr (D.-S.L.); hoh@wku.ac.kr (H.O.); Tel.: +82-63-230-6386 (D.-S.L.); +82-63-850-6815 (H.O.)
† These authors contributed equally to this work.

Contents

Figure S1. ^{1}H NMR spectrum (methanol-d_4) of epideoxybrevianamide E (**1**)

Figure S2. ^{13}C NMR spectrum (methanol-d_4) of epideoxybrevianamide E (**1**)

Figure S3. HRESI-MS spectrum of epideoxybrevianamide E (**1**)

Figure S4. ^{1}H NMR spectrum (methanol-d_4) of brevianamide V/W (**2**)

Figure S5. ^{13}C NMR spectrum (methanol-d_4) of brevianamide V/W (**2**)

Figure S6. HRESI-MS spectrum of brevianamide V/W (**2**)

Figure S7. ^{1}H NMR spectrum (methanol-d_4) of brevianamide K (**3**)

Figure S8. ^{13}C NMR spectrum (methanol-d_4) of brevianamide K (**3**)

Figure S9. HRESI-MS spectrum of brevianamide K (**3**)

Figure S10. ^{1}H NMR spectrum (methanol-d_4) of brevianamide Q (**4**)

Figure S11. ^{13}C NMR spectrum (methanol-d_4) of brevianamide Q (**4**)

Figure S12. HRESI-MS spectrum of brevianamide Q (**4**)

Figure S13. ^{1}H NMR spectrum (methanol-d_4) of brevianamide R (**5**)

Figure S14. ^{13}C NMR spectrum (methanol-d_4) of brevianamide R (**5**)

Figure S15. HRESI-MS spectrum of brevianamide R (**5**)

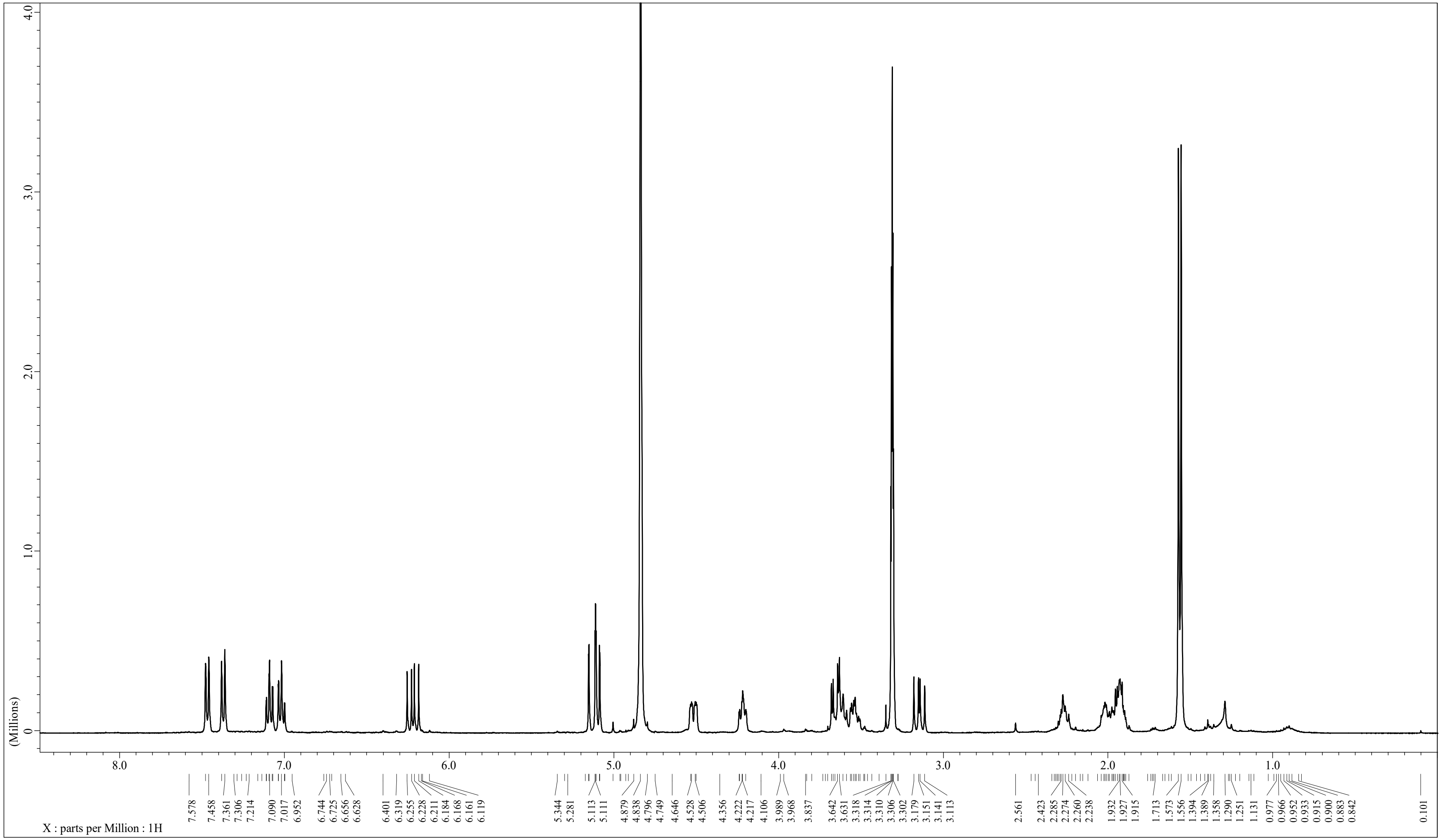

Figure S1. ^{1}H NMR spectrum (methanol-d_4) of epideoxybrevianamide E (**1**)

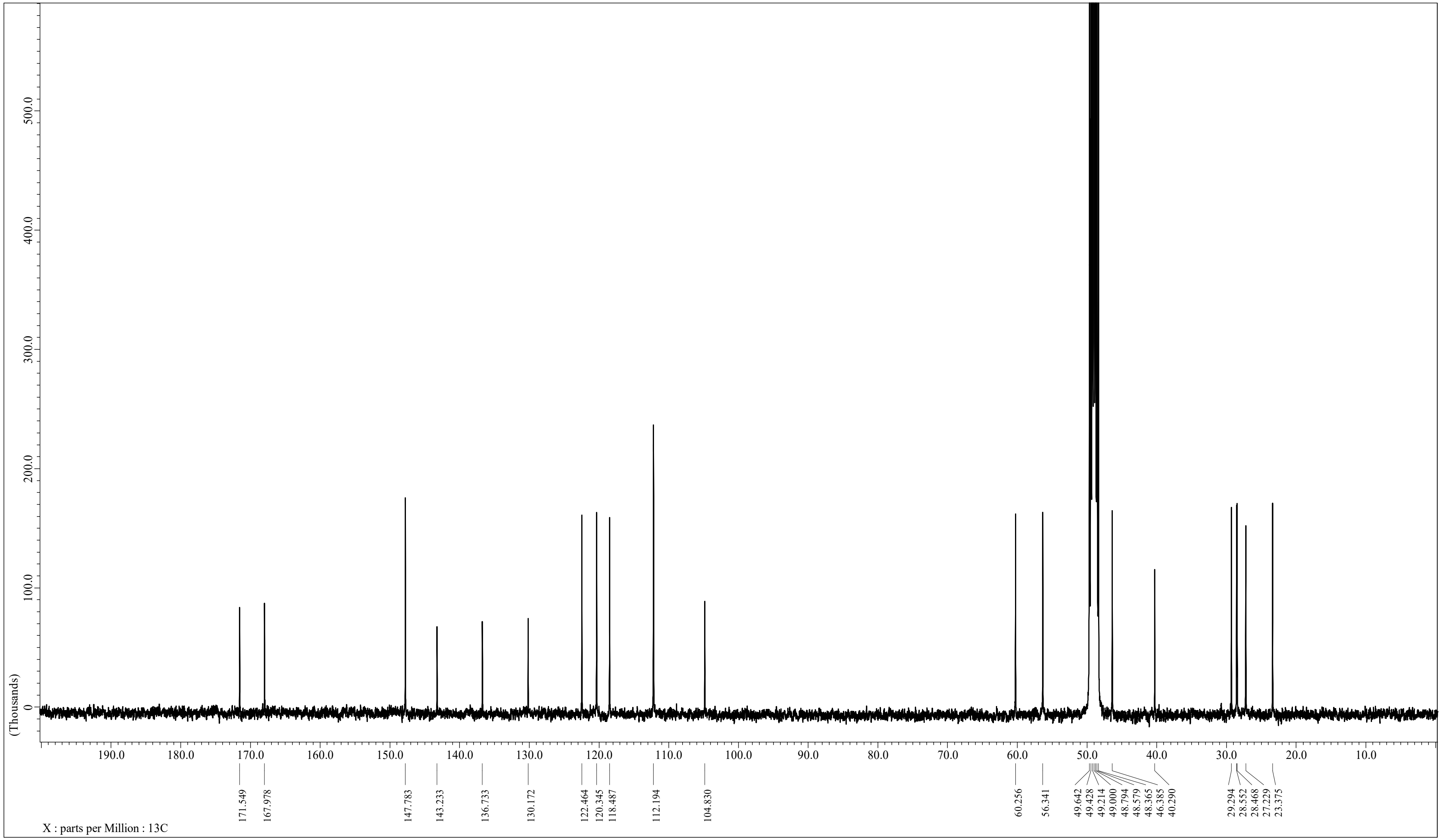

Figure S2. ^{13}C NMR spectrum (methanol-*d*$_4$) of epideoxybrevianamide E (**1**)

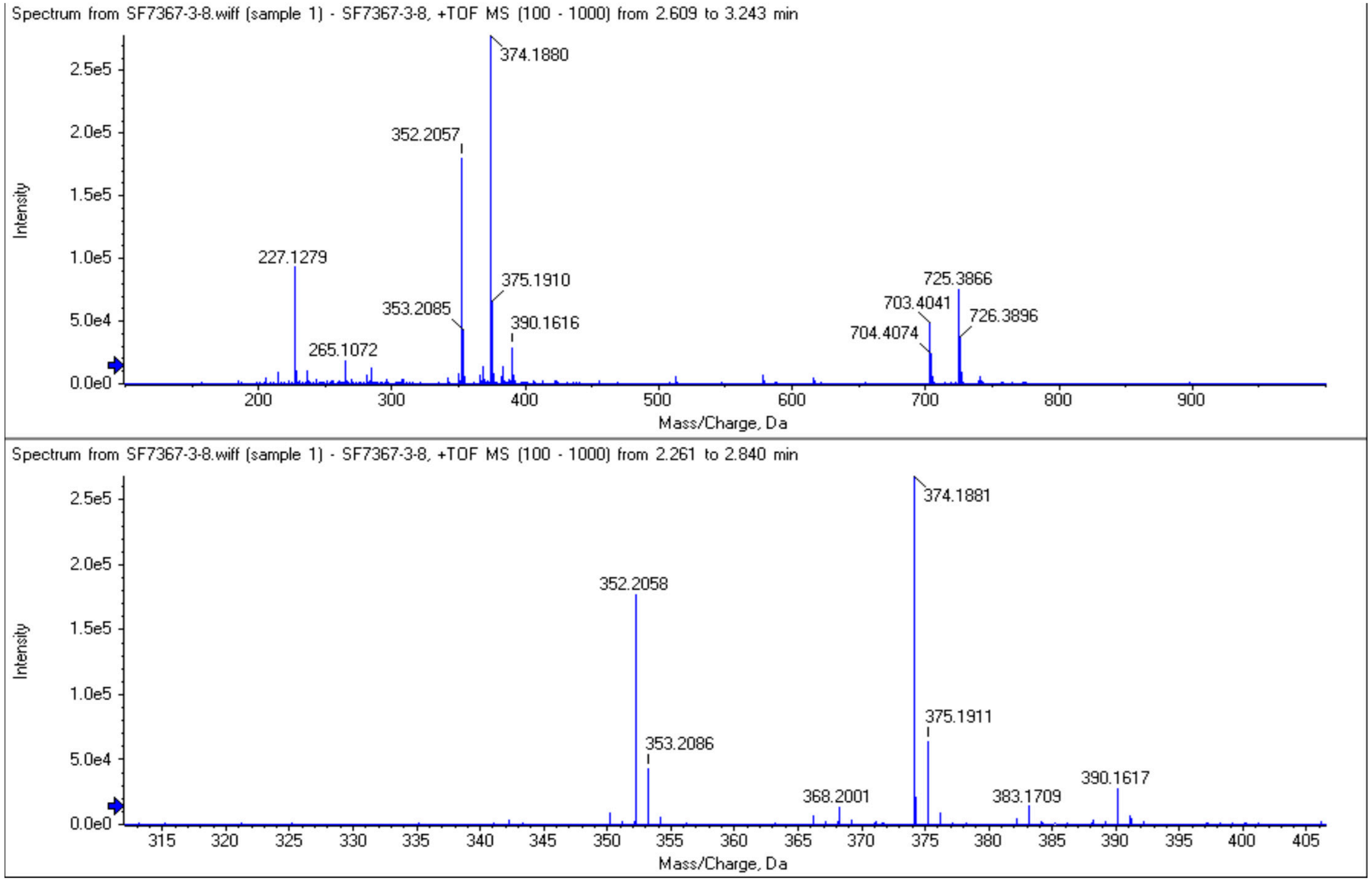

Figure S3. HRESI-MS spectrum of epideoxybrevianamide E (**1**)

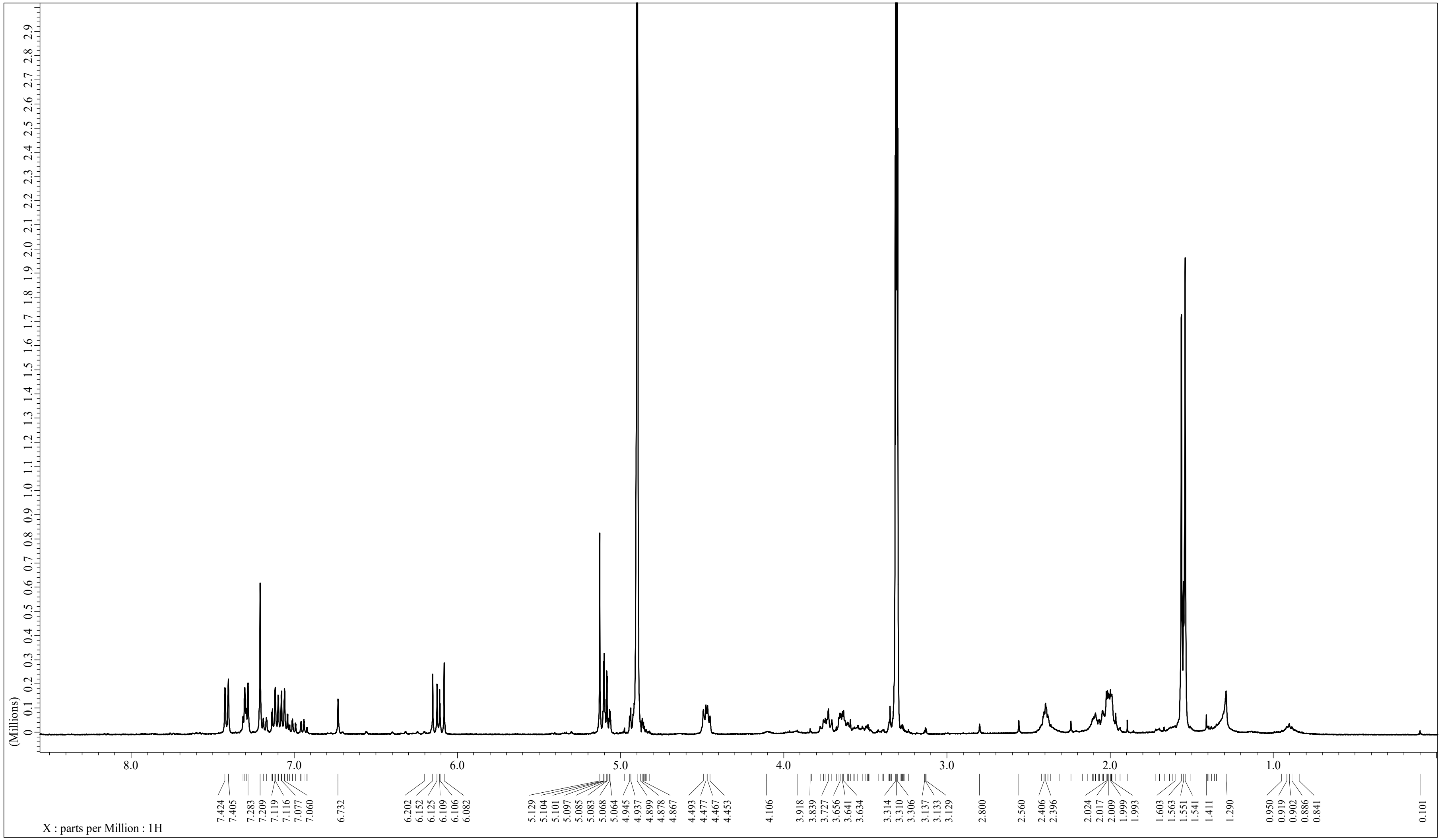

Figure S4. ^{1}H NMR spectrum (methanol-d_4) of brevianamide V/W (**2**)

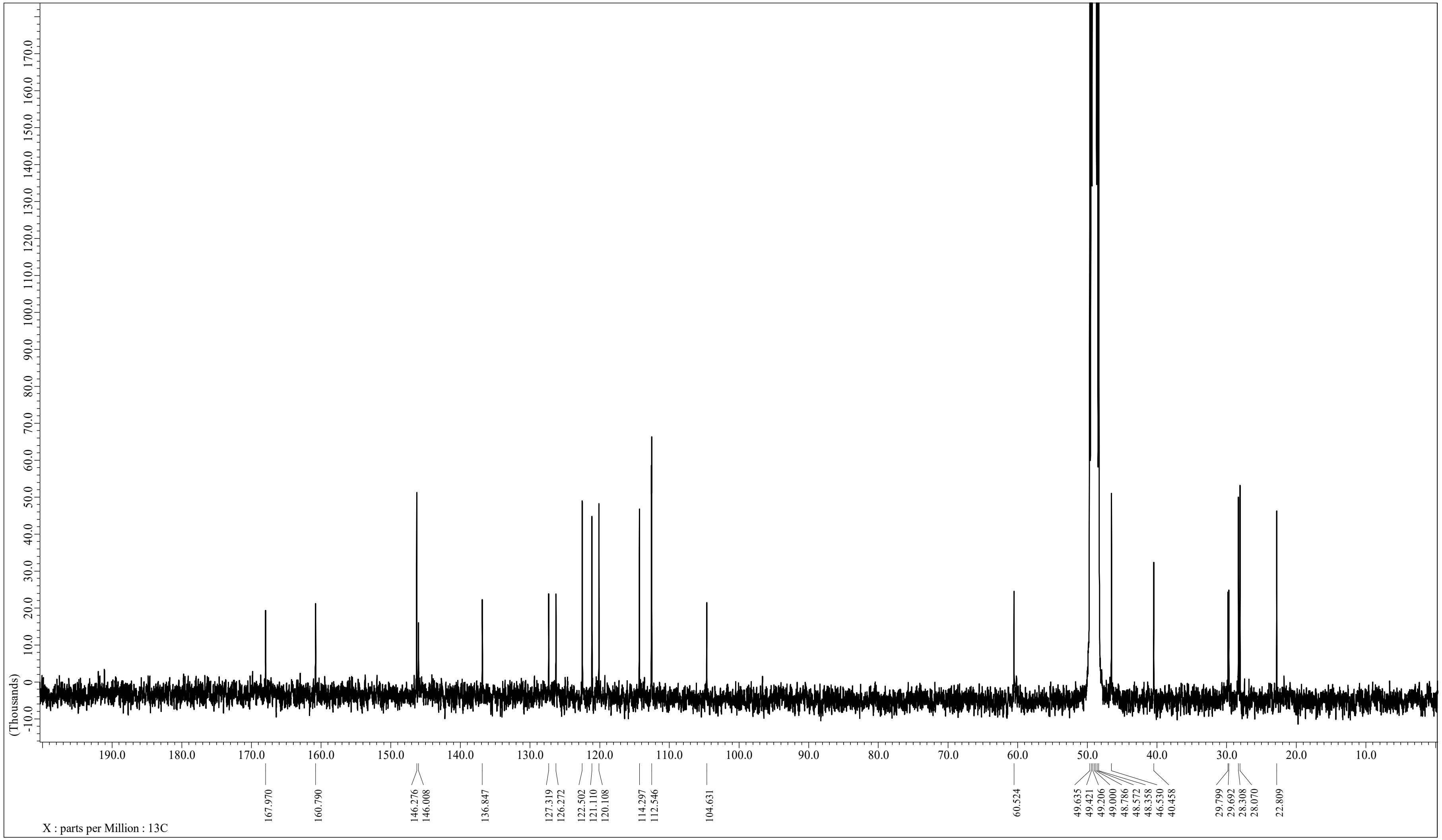

Figure S5. ^{13}C NMR spectrum (methanol-d_4) of brevianamide V/W (**2**)

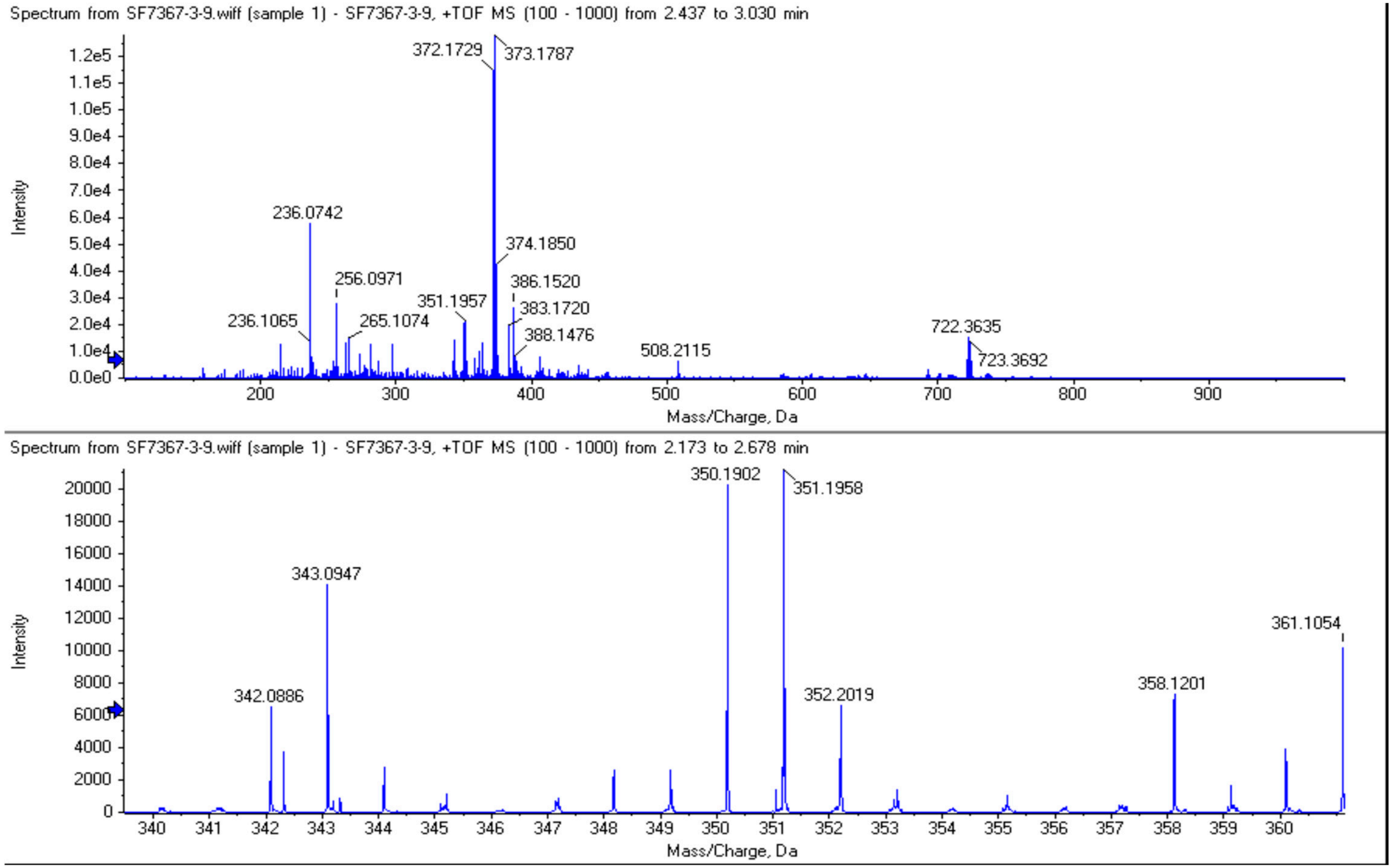

Figure S6. HRESI-MS spectrum of brevianamide V/W (**2**)

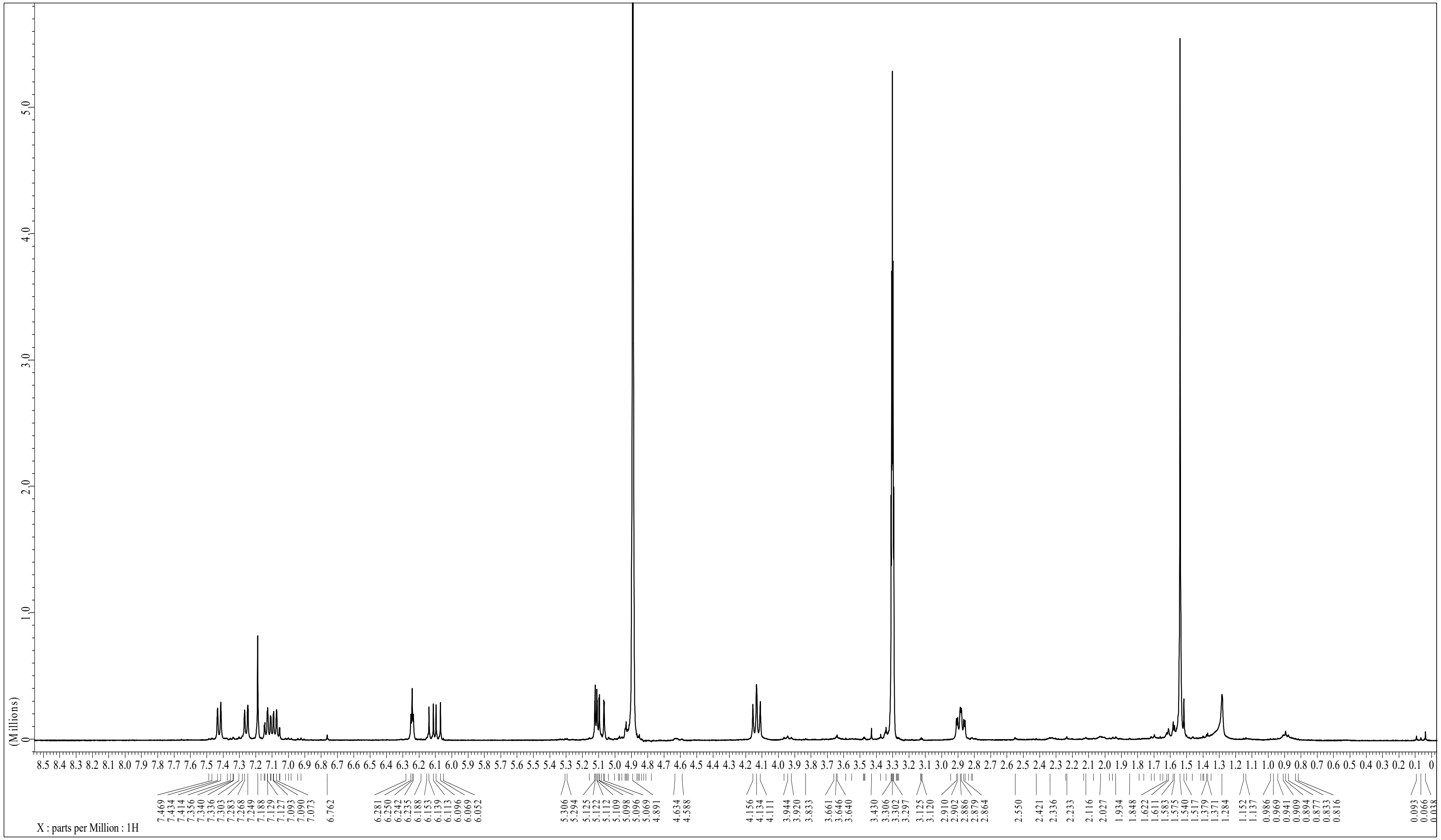

Figure S7. ^{1}H NMR spectrum (methanol-d_4) of brevianamide K (**3**)

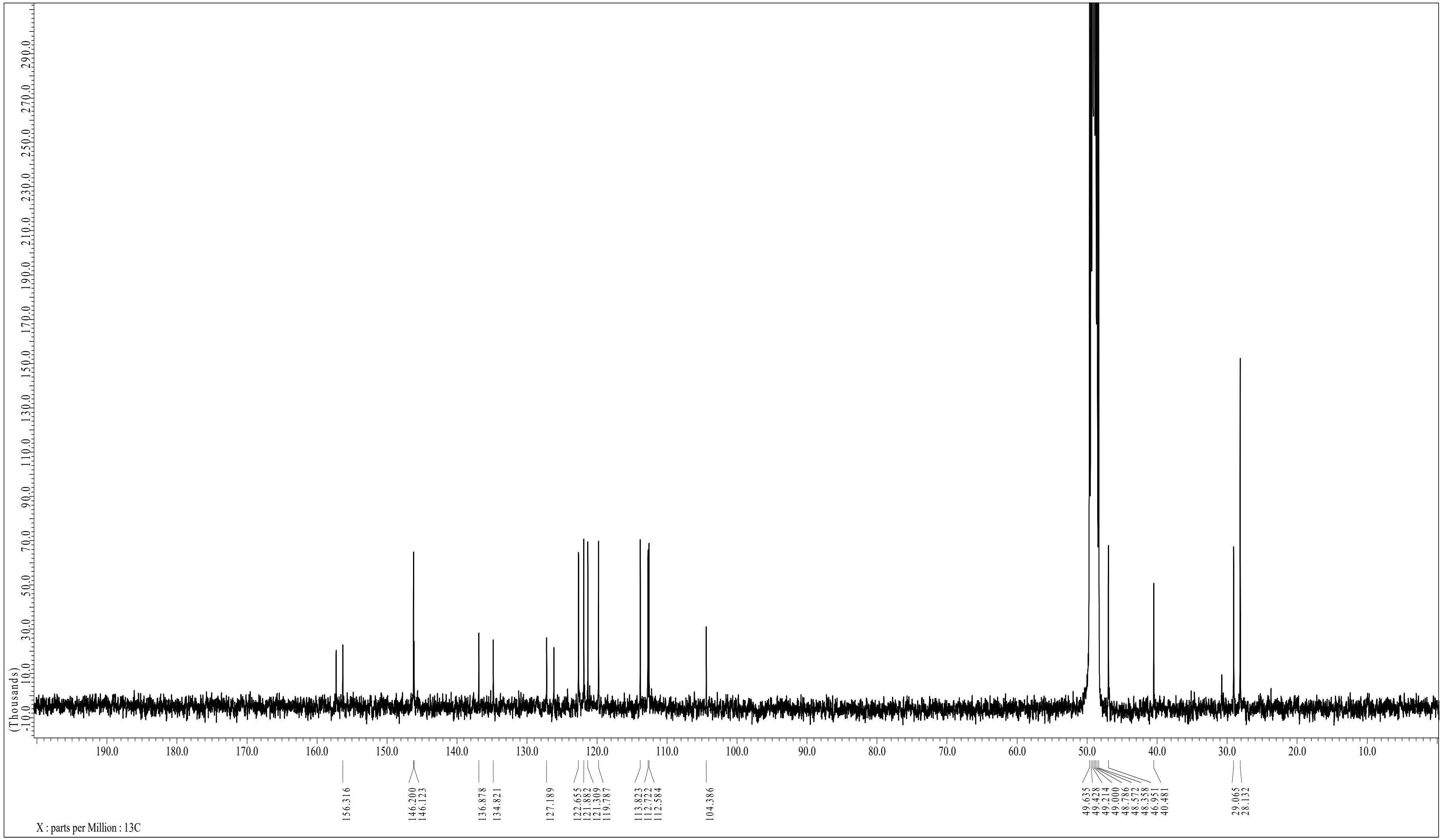

Figure S8. ^{13}C NMR spectrum (methanol-d_4) of brevianamide K (**3**)

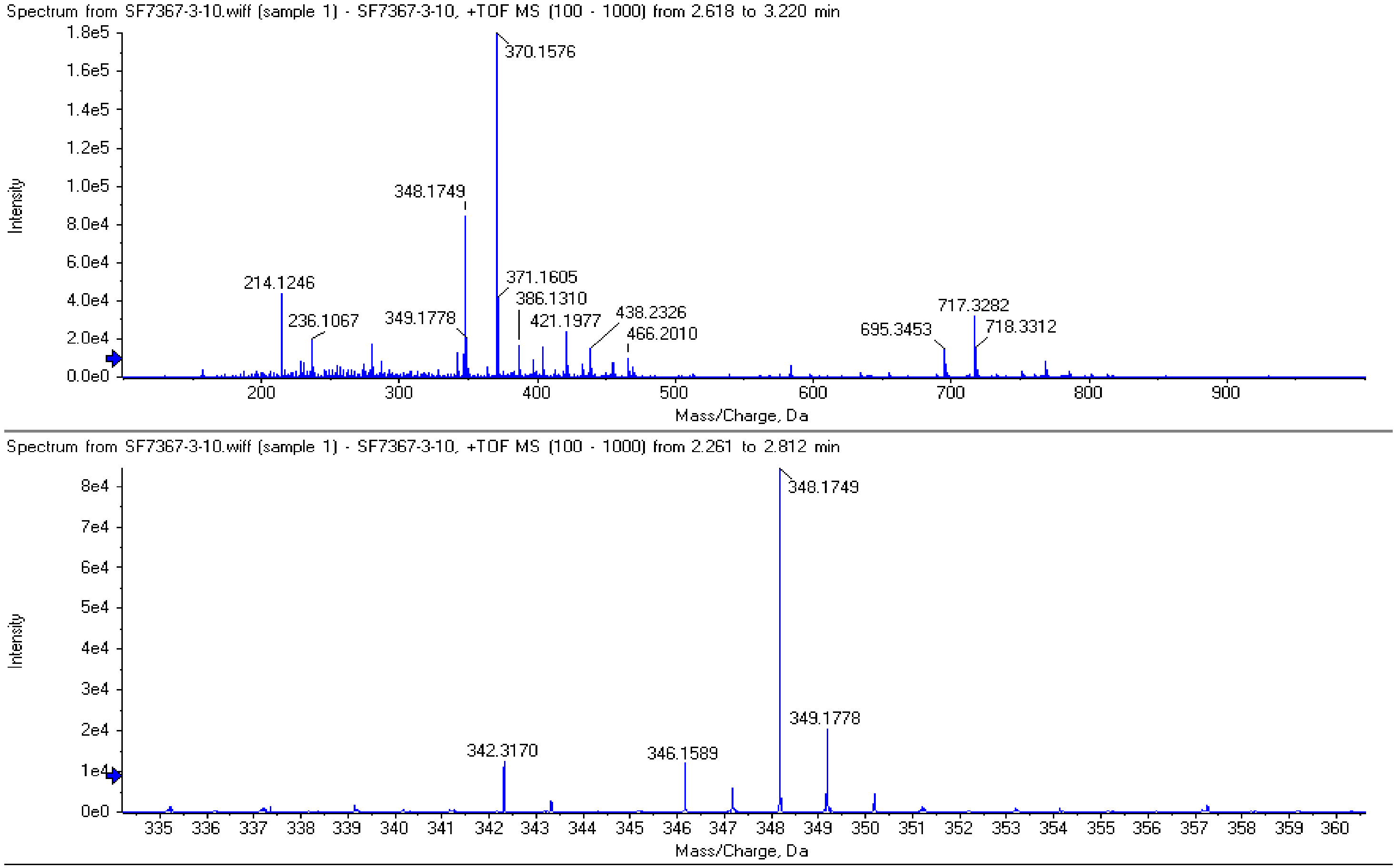

Figure S9. HRESI-MS spectrum of brevianamide K (**3**)

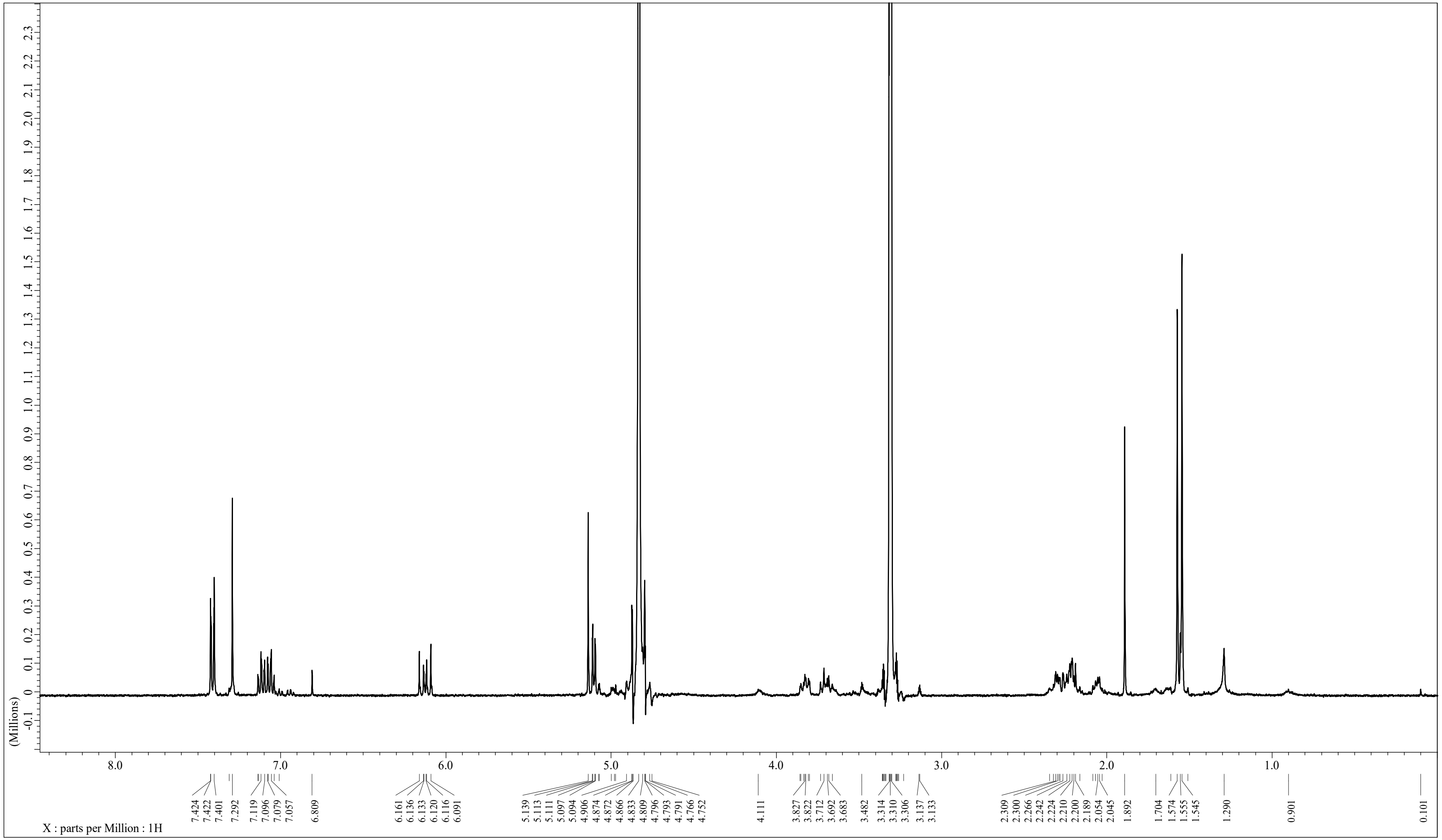

Figure S10. ^{1}H NMR spectrum (methanol-d_4) of brevianamide Q (**4**)

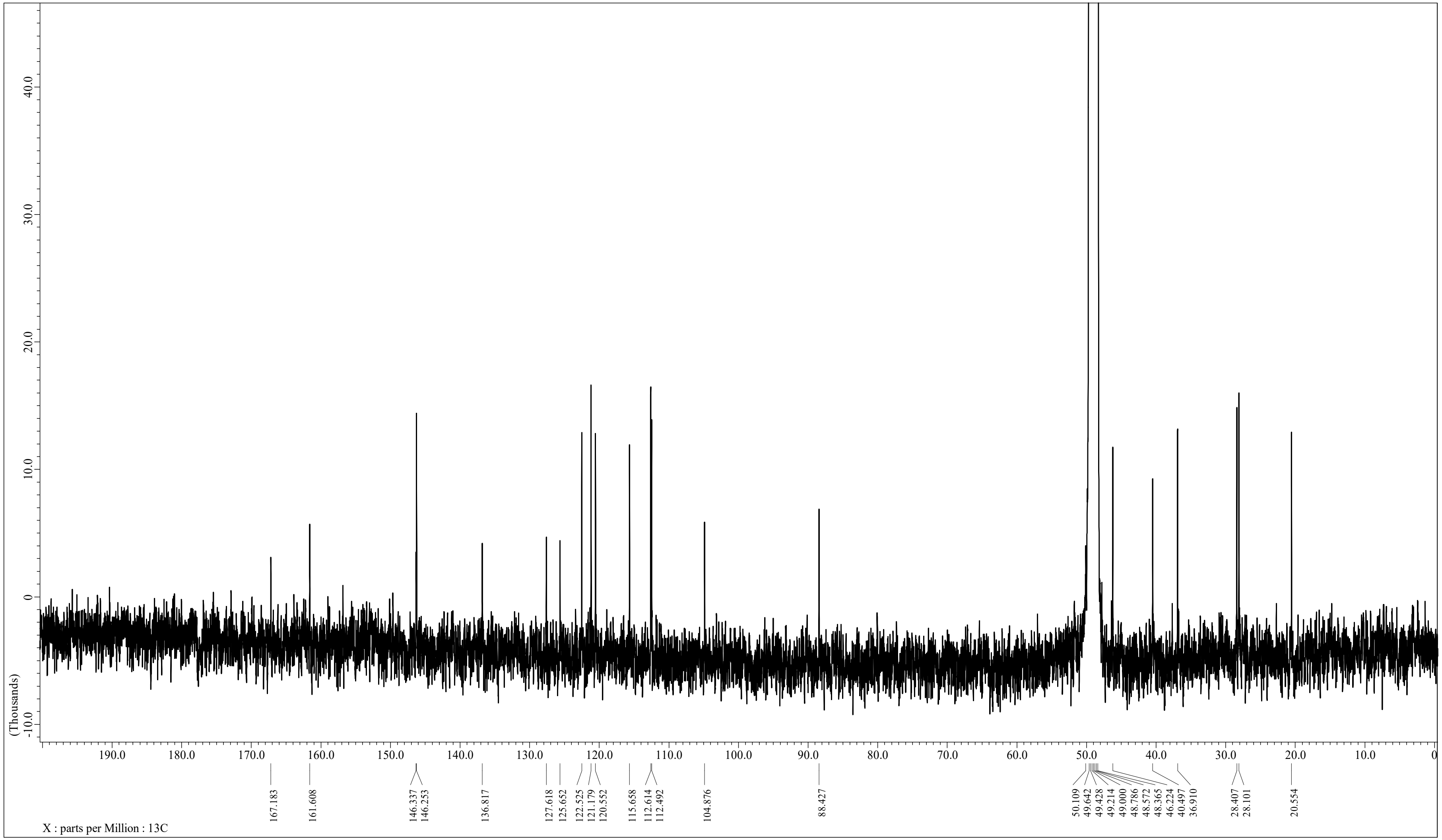

Figure S11. ^{13}C NMR spectrum (methanol-d_4) of brevianamide Q (**4**)

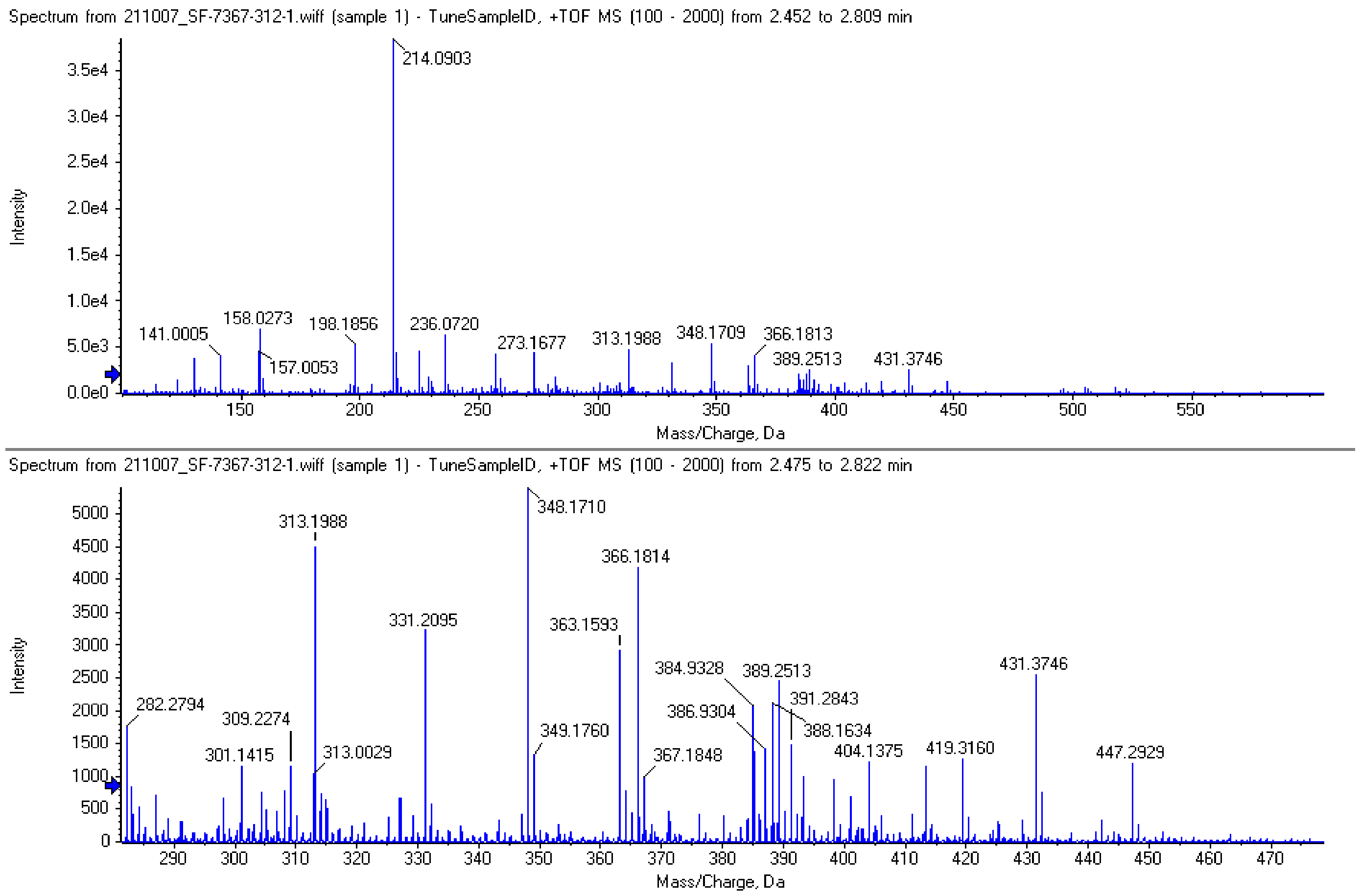

Figure S12. HRESI-MS spectrum of brevianamide Q (**4**)

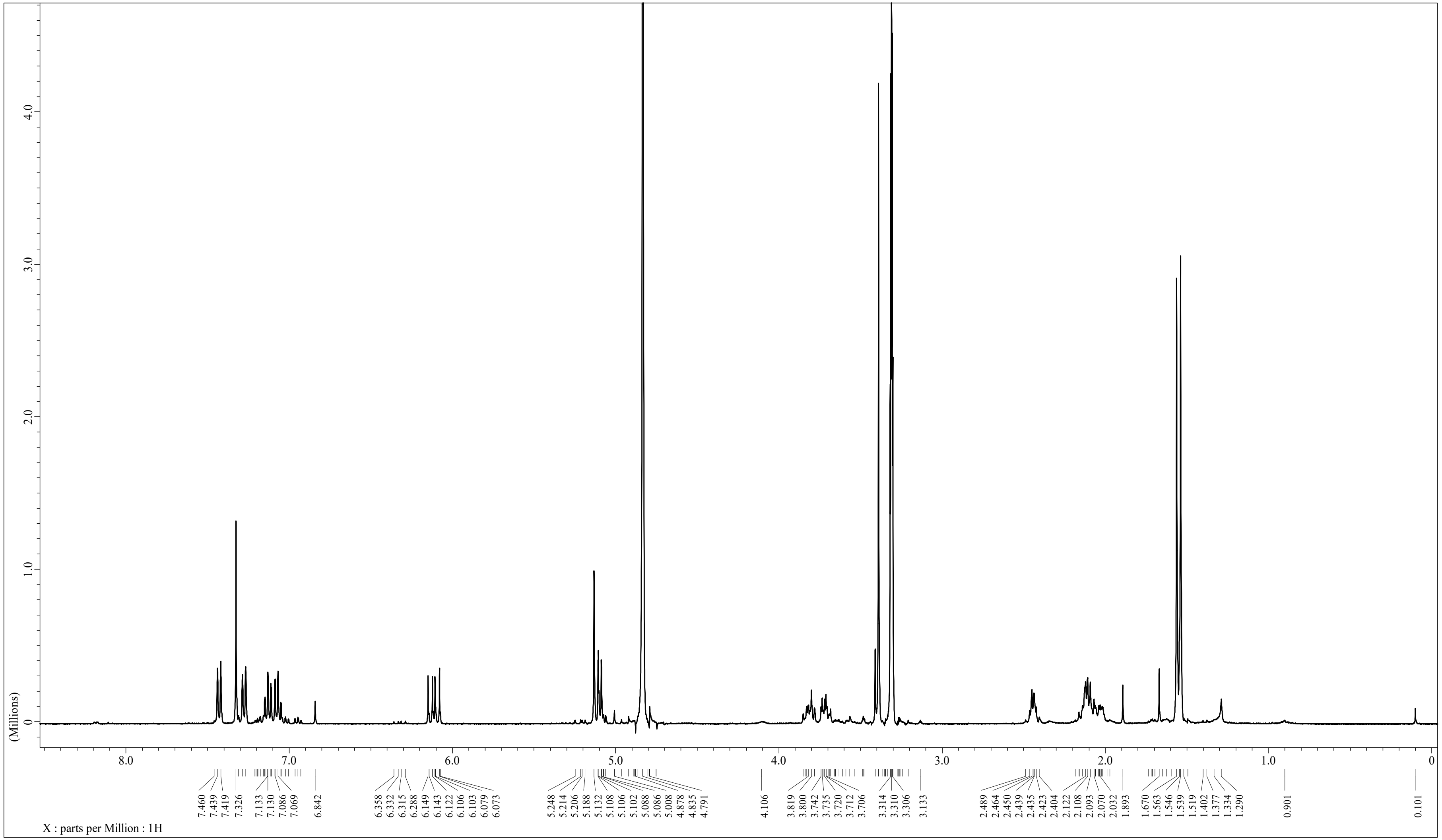

Figure S13. ^{1}H NMR spectrum (methanol-d_4) of brevianamide R (**5**)

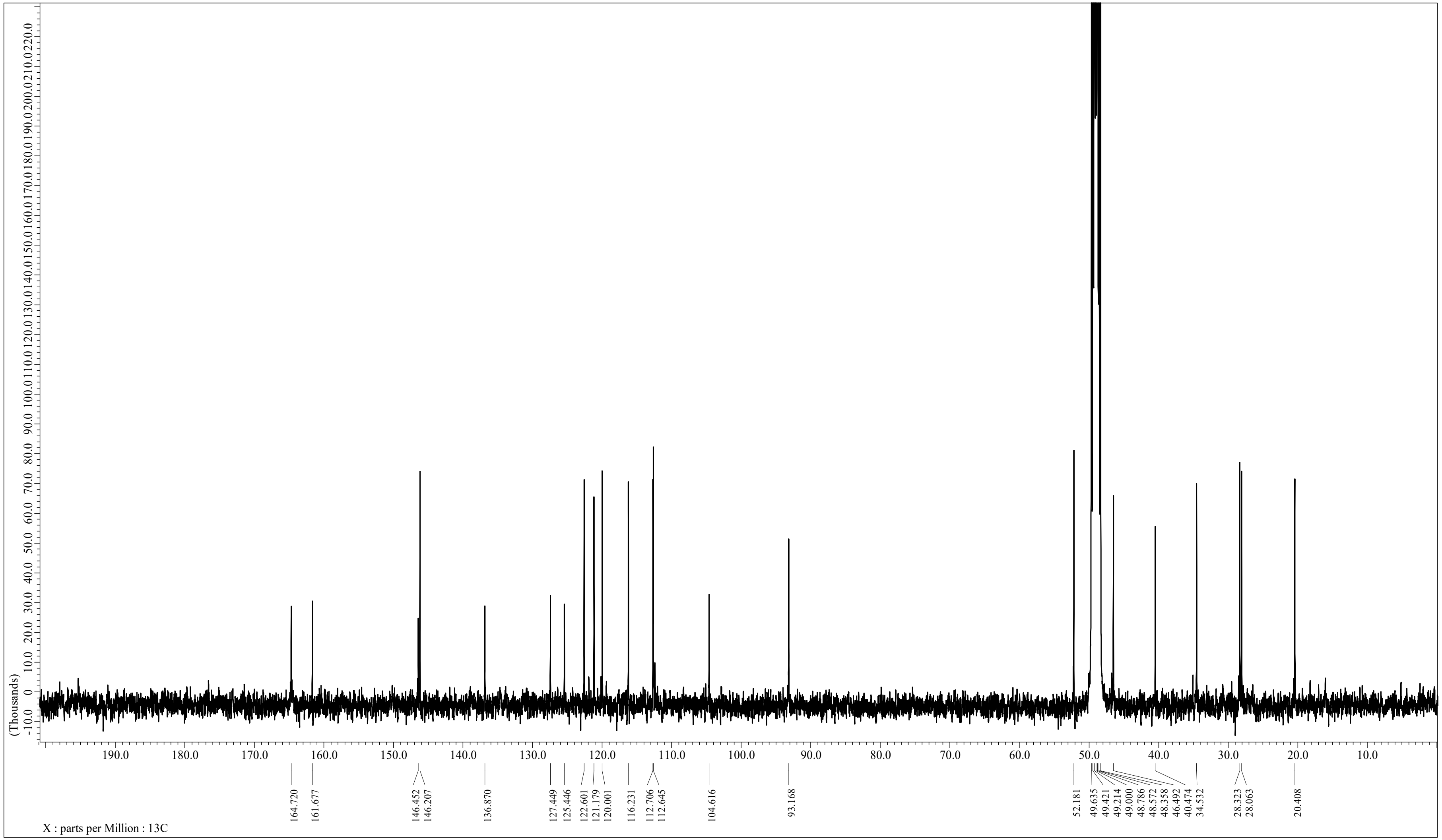

Figure S14. ^{13}C NMR spectrum (methanol-d_4) of brevianamide R (**5**)

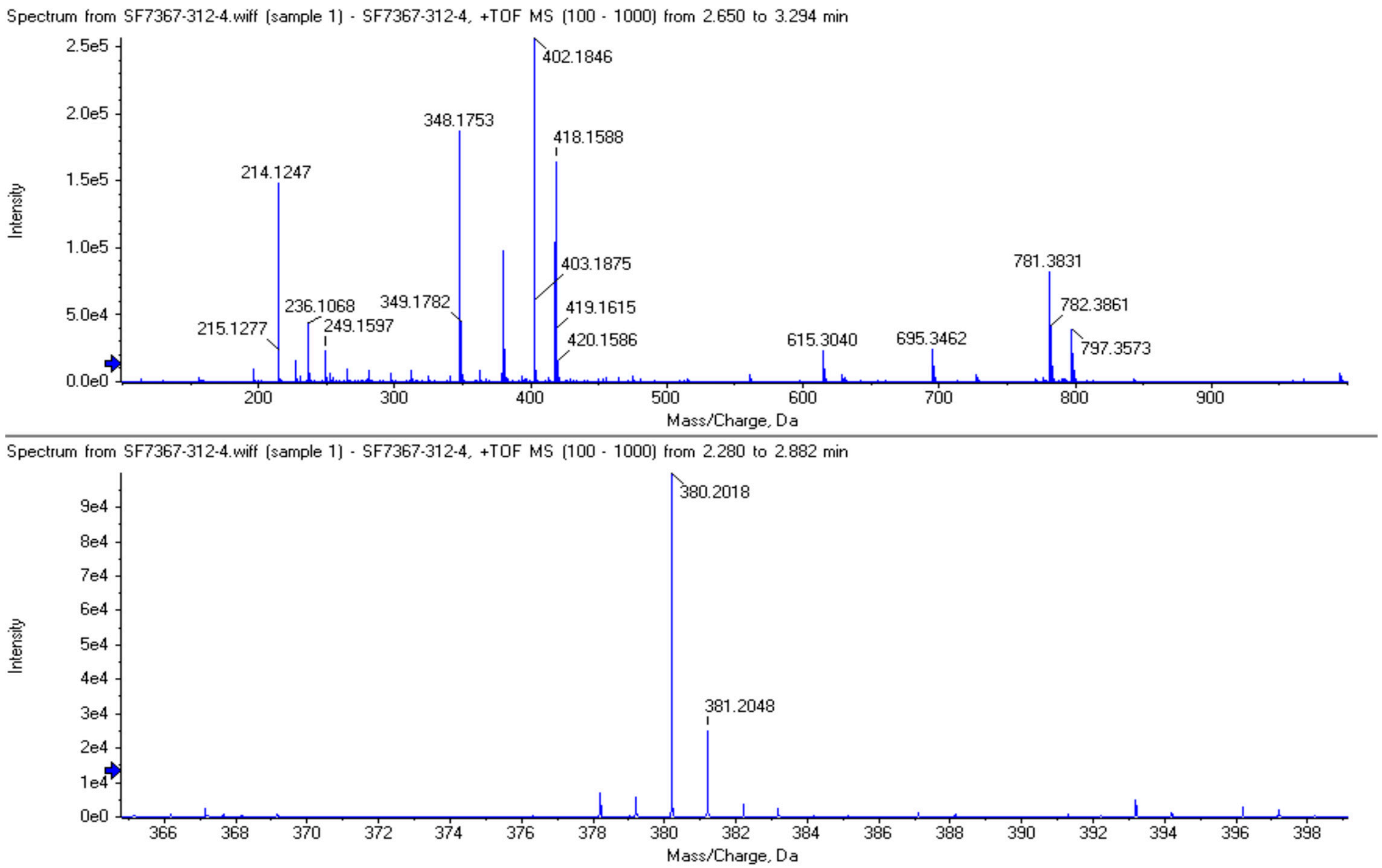

Figure S15. HRESI-MS spectrum of brevianamide R (**5**)
